# Long Term Suboxone™ Emotional Reactivity As Measured by Automatic Detection in Speech

**DOI:** 10.1371/journal.pone.0069043

**Published:** 2013-07-09

**Authors:** Edward Hill, David Han, Pierre Dumouchel, Najim Dehak, Thomas Quatieri, Charles Moehs, Marlene Oscar-Berman, John Giordano, Thomas Simpatico, Kenneth Blum

**Affiliations:** 1 Department of Software and Information Technology Engineering, École de Technologie Supérieure - Université du Québec, Montréal, Québec, Canada; 2 Department of Management Science and Statistics, University of Texas at San Antonio, San Antonio, Texas, United States of America; 3 Computer Science and Artificial Intelligence Laboratory, Massachusetts Institute of Technology, Cambridge, Massachusetts, United States of America; 4 Lincoln Laboratory, Massachusetts Institute of Technology, Lexington, Massachusetts, United States of America; 5 Occupational Medicine Associates, Watertown, New York, United States of America; 6 Departments of Psychiatry, Neurology, and Anatomy and Neurobiology, Boston University School of Medicine, and Boston Veteran Affaires Healthcare System, Boston, Massachusetts, United States of America; 7 G & G Holistic Health Care Services, LLC., North Miami Beach, Florida, United States of America; 8 Global Integrated Services Unit University of Vermont Center for Clinical and Translational Science, College of Medicine, Burlington, Vermont, United States of America; 9 Department of Psychiatry and McKnight Brain Institute, University of Florida, College of Medicine, Gainesville, Florida, United States of America; 10 Dominion Diagnostics, LLC, North Kingstown, Rhode Island, United States of America; 11 Department of Clinical Neurology, Path Foundation New York, New York, United States of America; 12 Department of Addiction Research and Therapy, Malibu Beach Recovery Center, Malibu Beach, California, United States of America; 13 Institute of Integrative Omics and Applied Biotechnology, Nonakuri, Purba Medinipur, West Bengal, India; UC Davis School of Medicine, United States of America

## Abstract

Addictions to illicit drugs are among the nation’s most critical public health and societal problems. The current opioid prescription epidemic and the need for buprenorphine/naloxone (Suboxone®; SUBX) as an opioid maintenance substance, and its growing street diversion provided impetus to determine affective states (“true ground emotionality”) in long-term SUBX patients. Toward the goal of effective monitoring, we utilized emotion-detection in speech as a measure of “true” emotionality in 36 SUBX patients compared to 44 individuals from the general population (GP) and 33 members of Alcoholics Anonymous (AA). Other less objective studies have investigated emotional reactivity of heroin, methadone and opioid abstinent patients. These studies indicate that current opioid users have abnormal emotional experience, characterized by heightened response to unpleasant stimuli and blunted response to pleasant stimuli. However, this is the first study to our knowledge to evaluate “true ground” emotionality in long-term buprenorphine/naloxone combination (Suboxone™). We found in long-term SUBX patients a significantly flat affect (p<0.01), and they had less self-awareness of being happy, sad, and anxious compared to both the GP and AA groups. We caution definitive interpretation of these seemingly important results until we compare the emotional reactivity of an opioid abstinent control using automatic detection in speech. These findings encourage continued research strategies in SUBX patients to target the specific brain regions responsible for relapse prevention of opioid addiction.

## Introduction

Substance seeking behavior has negative and devastating consequences for society. The total costs for drug abuse in the United States, is over $600 billion annually this includes lost productivity, health and crime-related costs, (*i.e.,* $181 billion for illicit drugs [1] $193 billion for tobacco [2], and $235 billion for alcohol [3]).

There has been a shift in mental health services from an emphasis on treatment focused on reducing symptoms based on health and disease, to a more holistic approach which takes into consideration quality of life [4]. Historically, the primary outcome goals for substance abuse treatment are harm reduction and cost effectiveness; with secondary outcomes including quality of life, and reduction of psychological symptoms [5]. Quality of life is characterized by feelings of wellbeing, control and autonomy, a positive self-perception, a sense of belonging, participation in enjoyable and meaningful activity, and a positive view of the future [4]. There is evidence that happy individuals are less likely to engage in harmful and unhealthy behaviors, including abuse of drugs and alcohol [5]. In addition, treatment approaches addressing depressive symptoms are likely to enhance substance-abuse treatment outcomes [6].

Opiate addiction, is a global epidemic, associated with many adverse health consequences such as fatal overdose, infectious disease transmission, and undesirable social consequences like, public disorder, crime and elevated health care costs [7]. Opioids have been implicated in modifying emotional states and modulating emotional reactions and have been shown to have mood-enhancing properties such as euphoria and reduced mood disturbance [8]. In methadone-maintained clients, the greatest reductions in mood disturbance correspond with times of peak plasma methadone concentrations [8]. Mood induction research suggests that methadone may blunt emotional reactivity [8]. Opioid users have abnormal emotional experience, characterized by heightened response to unpleasant stimuli and blunted response to pleasant stimuli [9]. There is evidence for a relationship between Substance Use Disorder and three biologically-based dimensions of affective temperament and behavior: negative affect (NA), positive affect (PA), and effortful control (EC). High NA, low EC, and both high and low PA were each found to play a role in conferring risk and maintaining substance use behaviors [10].

Buprenorphine/naloxone (Suboxone® [SUBX]) is used to treat opioid addiction because it blocks opiate-type receptors like mu, delta receptors and also provides agonistic activity [11], [12]. The Federal Drug Abuse Treatment Act of 2000 provides physicians who complete specialty-training to become certified to prescribe Suboxone® for treatment of opioid-dependent patients. Many clinical studies indicate that opioid maintenance with buprenorphine is as effective as methadone in reducing illicit opiate abuse while retaining patients in opioid treatment programs [13]. Diversion and injection of SUBX has been well documented [14]. Local, anecdotal reports are have been supported by recent international research which suggest that these medications also are used through other routes of administration, including smoking and snorting [15].

For the purpose of monitoring patients’ affective states, an area of growing interest is the understanding of changes in emotion during SUBX treatment. Although Blum and his colleagues suggested that long-term SUBX may result in anti-reward behavior coupled with affective disorders [16], there is little known concerning affect (“true” emotionality) in relation to actual reduction of Substance Use Disorder when patients are retained on SUBX during treatment.

To understand this relationship, and work toward employing an automatic means of monitoring patients’ emotions, we are investigating numerous previous speech classifier algorithms using Gaussian Mixture Modeling (GMM) [17]–[20]. In particular, the work from our laboratory by Sturim *et al.* [21] evaluated automatic detection of depression in speech and motivated the current research. We found in this earlier study of speech and depression that the introduction of a specific set of automatic classifiers (based on GMM and Latent Factor Analysis that recognized different levels of depression severity) significantly improved classification accuracy, over standard baseline GMM classifiers. Specifically Sturim *et al.* [21] saw large absolute gains of 20–30% Equal Error Rate for the two-class problem and smaller, but significant (approximately 10% Equal Error Rate) gains in two of the four-class cases. A detailed description of the core algorithm is provided in the Methods section of this article.

We monitored SUBX patients by using an evidence-based toolkit constructed from emotion detection in speech that can capture and accurately measure momentary emotional states of patients in their natural environment [22]–[24]. The benefits of this assessment toolkit, which includes the Experience Sample Method, are (1) collecting data on momentary states to avoid of recall deficits and bias, (2) ecological validity by data collection in the real-world, and (3) enabling analysis that is a dynamic process over time and can achieve temporal resolution. The Experience Sample Method is an excellent method for collecting data on participants’ momentary emotional states in their natural environment [25]. Based on the depressant pharmacological profile of opiate drugs, it seems reasonable to predict that SUBX patients would have flat affect and have low emotional self-awareness [8].

In this paper, we provide a qualification of emotional states followed by a description of empirical methods including subjects in this study, emotional state capture and measure, emotion detection in speech, calculation of emotional truth, and statistical analyses. Albeit needed opioid abstinent controls are absent, statistically significant results are presented to support and quantify the hypothesis that SUBX patients have a flat affect, have low emotional self-awareness and are unhappy.

“Affect” as defined by DSM-IV [26] is “a pattern of observable behaviors that is the expression of a subjectively experienced feeling state (emotion).” Flat affect refers to a lack of outward expression of emotion that can be manifested by diminished facial, gestural, and vocal expression.

Scott *et al.* [27] concluded that most chemically-dependent individuals have difficulty to identify their feelings and expressing them effectively. However, Scott *et al*. points out that they can change their responses to their emotions as they are better able to understand and tolerate their emotions [27]. Wurmser [28] coined the term “concretization” as the inability to identify and express emotions – a condition that often goes hand-in-hand with compulsive drug use. Wurmser further stated that it is as if these individuals have no language for their emotions of their inner life; they are unable to find pleasure in every-day life because they lack the inner resources to create pleasure.

Mood disorders (inappropriate, exaggerated, or limited range of feelings) and anxiety (stress, panic, agoraphobia, obsessive-compulsive, phobias) are directly associated with substance abuse. The National Epidemiologic Survey on Alcohol and Related Conditions performed a survey of 43,093 respondents [29]. Among respondents with any drug use disorder who sought treatment, 60.31% had at least one independent mood disorder, 42.63% had at least one independent anxiety disorder, and 55.16% had a comorbid alcohol use disorder. Of the 40.7% of respondents with an alcohol use disorder, had at least comorbid mood disorder while, more than 33% had one current anxiety disorder.

Dodge [6] concluded that higher depressive symptom scores significantly predicted and decreased the likelihood of abstinence after discharge from treatment centers, regardless of type of substance abuse, the frequency of substance use, or length of stay in treatment. Dodge further stated that treating the depressive symptoms could enhance outcomes in substance-abuse treatment.

According to Fredrickson’s [30] broaden-and-build theory, the essential elements of optimal functioning are multiple, discrete, positive emotions and the best measure of ^“^Objective happiness" is tracking and later aggregating people's momentary experiences of good and bad feelings. The overall balance of people's positive and negative emotions has been shown to predict their judgments of subjective well-being.

Lyubomrsky *et al*. [31] determined that frequent positive affect as a hallmark of happiness has strong empirical support. Whereas the intensity of emotions was a weak indicator of self-reports of happiness, a reliable indicator was the amount of time that people felt positive emotions relative to negative emotions. High levels of happiness are reported by people who have predominantly positive affect, 80% or more of the time. There might be a connection between positive emotions and willpower, and the ability to gain control over unhealthy urges and addictions.

Tugade *et al*. [32] determined that the anecdotal wisdom, that positive emotions are beneficial for health is substantially supported by empirical evidence. Those who used greater proportion of positive rather than negative emotional words showed greater positive morale and less depressed mood.

With respect to the findings in this study, the subject’s momentary emotional states were set to include the actual emotion expressed by the individual (henceforth “emotional truth”), emotion expressiveness, ability to identify one’s own emotion (henceforth “self-awareness”), and the ability to relate to another person’s emotion (henceforth “empathy”).

## Methods

### Subjects

This project originated from the department of Software Engineering and Information Technologies at École de Technologie Supérieure (ETS), a division of University of Quebec. This project was approved by the Board of Ethics at École de Technologie Supérieure, University of Quebec. A consent form, approved by the University of Quebec Ethics Committee (Canadian equivalent to the American IRB informed consent), was signed by each participant. We did not ask participants any information other than gender and language due to ethics committee restrictions (see Table 1). Therefore, this impeded specific demographic elements with regard to all participants in this study. Statistical analyses were conducted in the autumn of 2011, in preparation for presentations to psychologists at the Center for Studies on Human Stress in Montreal. The 36 SUBX patients were randomly urine screened for the presence of SUBX. The urine screening revealed the presence of SUBX in 100% of these patients. Testing was performed by off-site by Quest Diagnostics (727 Washington St., Watertown, NY 13601, USA), and on-site at Occupational Medicine Associates of Northern New York, using the Proscreen drug test kit provided by US Diagnostics (2007, Bob Wallace Avenue, Huntsville, AL 35805, USA).

**Table 1:** Gender and language of the research participants.

Group	Females	Males	English	French
**General Population (GP)**	29	15	25	19
**Alcoholics Anonymous (AA)**	4	29	33	0
**Suboxone (SUBX)**	23	13	36	0
**Totals**	56	57	94	19
	113		113	

### Emotional State Capture and Measure

We use an Interactive Voice Response system that called patients on their telephone in their natural environment, collected momentary emotional states in a 15-second dialogue that reduces subject burden typical of pen-and-pencil journaling and mobile applications. The emotion class set that we selected (Neutral, Happy, Sad, Angry and Anxious) covered the key drug use mood disorders of anxiety and depression. Affect neutrality captures emotional states including calmness, feeling “normal” or “okay,” and contentment. Happiness is linked to abstinence [6]. We selected a maximum of five choices in our interactive dialog with patients, to conform to Miller’s [33] model that the forward short-term memory span of human is 7±2. Additionally, the INTERSPEECH 2009 Emotion Challenge largest classification set of emotions contained the following categories: Angry, Emphatic, Neutral, Positive, and Rest [23].

113 trial participants received 19,539 telephone calls in data collection trials held in 2010 and 2011. Calls were placed on a daily basis at a time of day chosen by the participant. Of these placed phone calls, a total of 8,376 momentary emotional states were collected. 11,163 calls were automatically aborted due to a busy signal, no answer, or voice mail answer. The 113 participants included three groups: General Population (GP), N = 44 [15 men; Expressions = 2,440]; Alcohol Anonymous (AA), N = 33 [29 men; Expressions = 3,848]; and SUBX, N = 36 [13 men; Expressions = 1054] with an average SUBX continued maintenance period of 1.66 years (SD = 0.48). All three groups were included in the statistics, and all results were statistically significant (p<0.05) except for trends (p<0.1) with regard to happiness self-awareness (defined below) derived from self-report emotion measurements.

**Figure 1:**
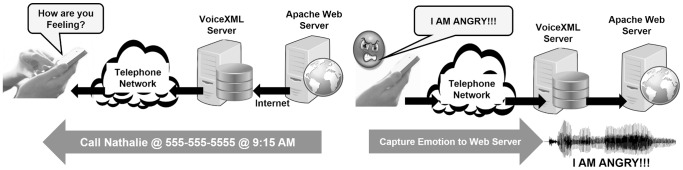
Patient Momentary Emotional State collection through the Interactive Voice Response system. Patient-reported-outcome (PRO) Experience Sampling Method (ESM) data collection places considerable demands on participants. Success of an ESM data collection depends upon participant compliance with the sampling protocol. Participants must record an ESM at least 20% of the time when requested to do so; otherwise the validity of the protocol is questionable. The problem of “hoarding” – where reports are collected and completed at a later date – must be avoided. Stone et al confirmed this concern through a study and found only 11% of pen-and-pencil diaries where compliant; 89% of participants missed entries, or hoarded entries and bulk entered them later. [58] IVR systems overcome hoarding by time-sampling and improve compliance by allowing researchers to actively place outgoing calls to participants in order to more dynamically sample their experience. Rates of compliance in IVR sampling literature vary from as high as 96% to as low as 40% [59] Subject burden has also been studied as a factor effecting compliance rates. At least six different aspects affect participant burden: Density of sampling (times per day); length of PRO assessments; the user interface of the reporting platform; the complexity of PRO assessments (i.e. the cognitive load, or effort, required to complete the assessments); duration of monitoring; and stability of the reporting platform [59]. Researchers have been known to improve compliance through extensive training of participants [58]. Extensive training is impractical for automated ESM systems. Patients were called by the IVR system at designated times thus overcoming hoarding. A simple intuitive prompt: “How are you feeling?” elicited emotional state response (e.g., “I am angry!”); no training was required. The audio response is recorded on the web server for analysis. The IVR system was implemented through the W3C standards CCXML and VoiceXML on a Linux-Apache-MySQL-PHP (LAMP) server cluster.

**Figure 2:**
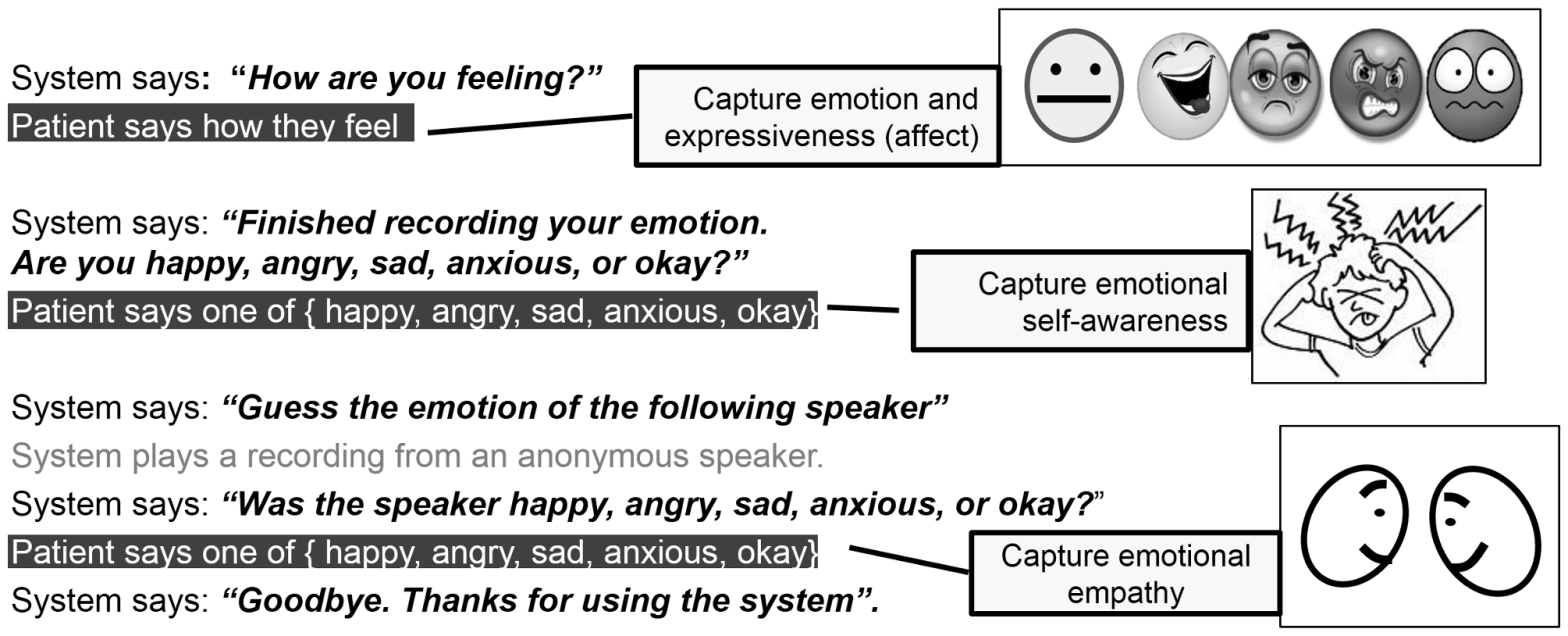
An Interactive Voice Response dialogue. The Voice User Interface (VUI) dialogue was carefully crafted to (1) capture a patient’s emotional expression, emotional self-assessment, and empathic assessment of another human’s emotional expression; and (2) to avoid subject-burden and training. The average call length is 12 seconds thus alleviating subject-burden (post collection surveys indicate ease-of-use. Call completion rates were 40% (95% CI: 33.6–46.7) (p = 0.003). Emotional expression in speech is elicited by asking the quintessential question “how do you feel?” It is human nature to colour our response to this question with emotion [27]. Emotional self-assessment is captured by asking the patient to identify their emotional state from the emotion set: (Neutral, Happy, Sad, Angry and Anxious) by selecting the corresponding choice on their DTMF telephone keypad. The system captures empathy by prompting the patient with: “guess the emotion of the following speaker” followed by the playback of a randomly selected previously captured speech recording from another patient. The patient listens to the emotionally charged speech recording and registers an empathy assessment by selecting the corresponding choice from the emotion set on their DTMF telephone keypad.

As can be seen in Figure 1, the participants were prompted with “How are you feeling?” and the audio response (e.g., “I am angry!”) was recorded on the web server [24]. The entire 15-second dialogue is depicted in Figure 2. The emotional truth of the audio response to “How are you feeling?” was measured and classified within the set of Neutral, Happy, Sad, Angry, and Anxious. Expressiveness was measured from the emotional-truth calculation’s confidence score and the length of speech (described in the emotional-truth calculation section). Self-awareness was computed by comparing the emotional truth to the patient’s self-assessment, which was captured in response to the prompt “Are you happy, angry, sad, anxious or okay?” Empathy was computed by comparing the patient’s response to the anonymous recording following the prompt “Guess the emotion of the following speaker” to the emotional truth of that same anonymous recording.

**Figure 3:**
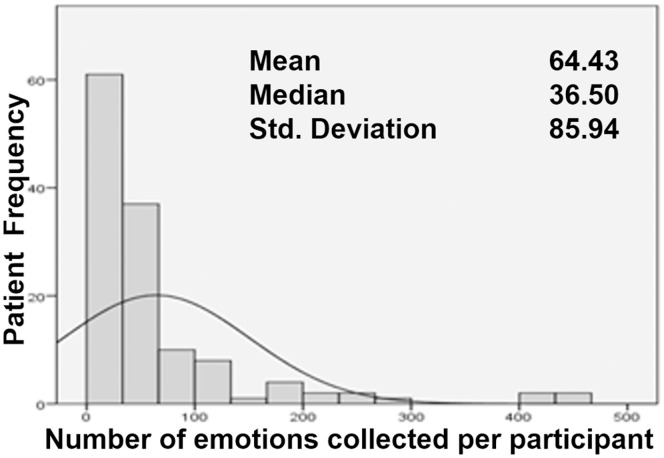
Frequency of emotional states collected per participant. Trial data capture is multilevel with emotional state samples grouped within patients. Frequencies of samples per patient are skewed towards a Poisson distribution; typical of ESM data collections. The mean is 64.4 and the median is 36.5 momentary emotional states per patient. On average participants answered 41% of emotion collection calls. SUBX patients answered significantly fewer calls (18.6%) as compared to the General Population (56.4%) and members of Alcoholics Anonymous (49.3%).

**Figure 4:**
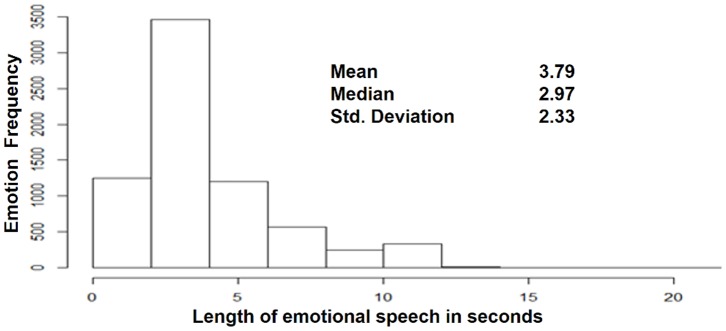
Speech duration of emotional responses. Speech duration of patients’ emotional expression in response to “How are you feeling?” shows that 75% of speech captured was less than 4.6 seconds.The mean is 3.79 seconds and the median is 2.97 seconds. Some utterances (e.g. “ok”) are as short as 0.1 seconds, Minimum and maximum speech durations influenced the design of the speech activity detector.

Frequencies in Figure 3 describe and graph the momentary emotional states collected per trial participant. Frequencies were skewed towards a Poisson distribution. The median was 36.5 momentary emotional states collected per participant. Figure 4 depicts the frequencies of the speech duration in response to “How are you feeling?” Most speech captured was less than 5 seconds in duration.

### Emotion Detection

**Figure 5:**
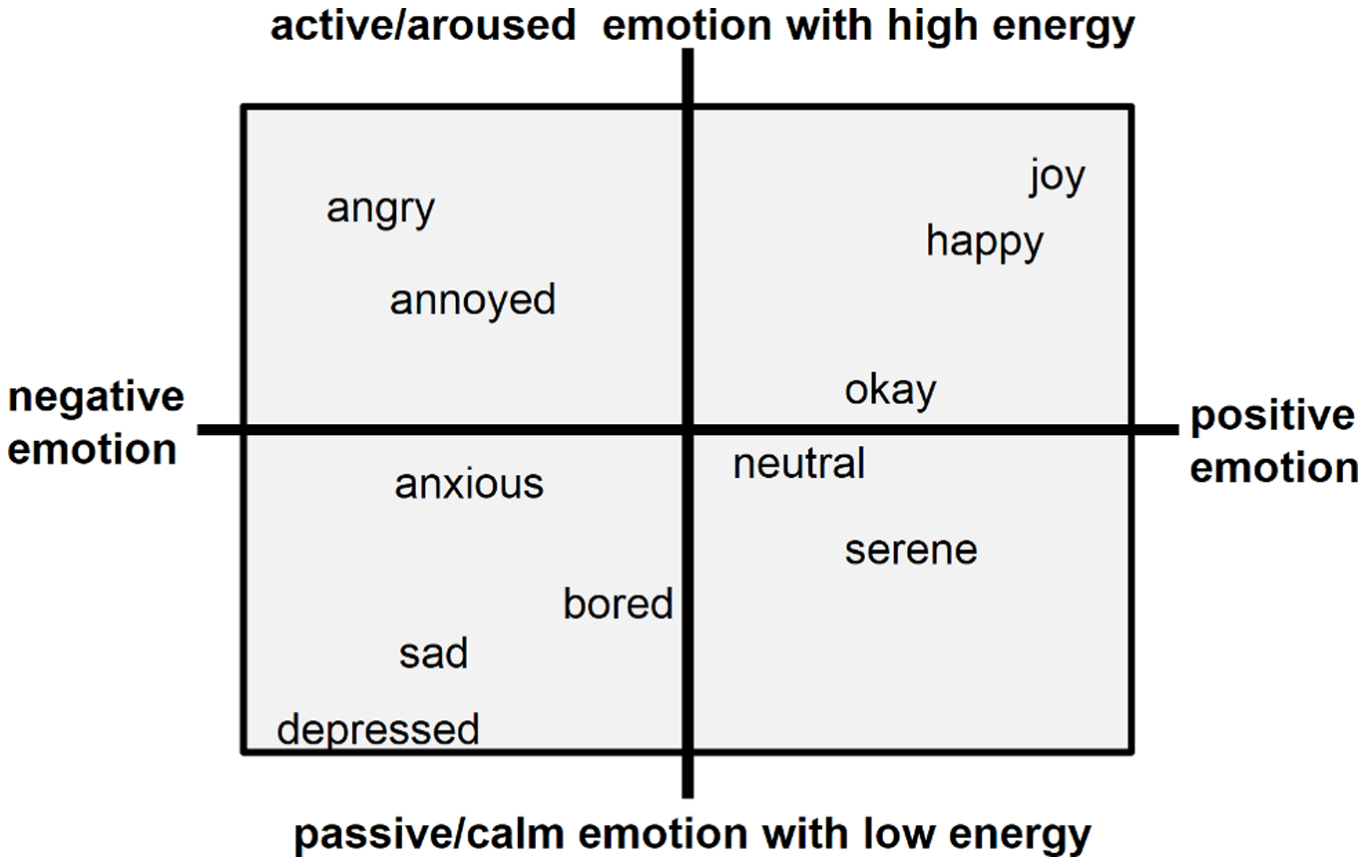
Activation-Evaluation Emotional space. The activation dimension (a.k.a. arousal dimension) refers to the degree of intensity (loudness, energy) in the emotional speech; and the evaluation dimension refers to how positive or negative the emotion is perceived [37]. Emotional states with high and low level of arousal are hardly ever confused, but it is difficult to determine the emotion of a person with flat affect [36]. Emotions that are close in the activation-evaluation emotional space (flat affect) often tend to be confused [37].

The desired approach to emotional truth determination is automatic real-time emotion detection in speech. The core algorithm of an emotion detector in speech has been developed through a collaborative of scientists from the Massachusetts Institute of Technology (MIT) and the University of Québec. The automatic classifier filters silence and non-speech from the recording, computes Mel-Frequency Cepstral Coefficient (MFCC) and energy features from filtered speech, and then classifies these features to an emotion. Gaussian Model Mixtures (GMMs) were trained for each emotion in the set (Neutral, Happy, Sad, Angry and Anxious). The training data were labeled with a fused weighted majority vote classifier [34] including professional transcribers, anonymous voters, and self-assessment (described in the emotional truth section – excluding the emotion detector). The maximum likelihood of the emotion contained in the speech was then computed using the trained GMMs [17]–[23], [35]. In determining emotion characteristics, we note that, in a post-trial survey, 85% of trial participants indicated they listened to how the speaker spoke, rather than what was said, to determine emotion. Emotional states with high and low level of arousal are hardly ever confused, but it is difficult to determine the emotion of a person with flat affect [36]. Emotions that are close in the activation-evaluation emotional space (flat affect) often tends to be confused [37] (see Figure 5). Steidl *et al*. found that, in most cases, three out of five people could agree on the emotional content [38]. Voter agreement is, therefore, an indication of emotion confusability and flat affect. The ratio of votes that are in agreement is a confidence score of the emotional truth.

Our approach to automatic emotion detection in speech is inspired from Dumouchel *et al*. [22], [39] and consists of extracting Mel-Frequency Cepstral Coefficients (MFCCs) and energy features from speech and then classifying these acoustic features to an emotion. A large GMM referred to as the Universal Background Model, which plays the role of a prior for all emotion classes, was trained on the emotional corpus of 8,376 speech recordings using the Expectation-Maximization algorithm. After training the Universal Background Model, we adapted it to the acoustic features of each emotion class using the Maximum A posteriori (MAP) algorithm. As in Reynolds *et al*. [40] we used MAP adaptation rather than the classic Maximum Likelihood algorithm because we had very limited training data for each emotion class (which increased the difficulty of separate training of each class GMM).

**Figure 6:**
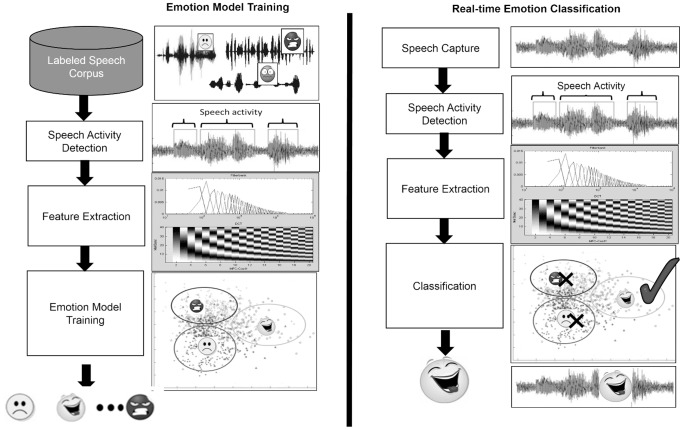
Two stages of emotion detection: model training and real time detection. Figure 6 depicts the model training and detection stages of the emotion detector. Models are trained in the left pane of the figure. Emotion detection classification computes the most likely emotion using the trained models as shown in the right pane. Speech Activity Detection and Feature Extraction are identical in both model training and classification.

Figure 6 depicts the model training and detection stages of the emotion detector. Models are trained in the left pane of the figure. Emotion detection classification computes the most likely emotion using the trained models as shown in the right pane. Speech Activity Detection and Feature Extraction are identical in both model training and classification.

The Speech Activity Detector [41] removes the silence and non-speech segments from the recording prior to feature extraction. Experiments were performed to optimize parameters with the goal of ensuring no valid speech recordings were discarded (e.g., the response utterance “ok” can be as short as 0.2 seconds), and the GMM emotion detector’s accuracy was maximized.

MFCCs were calculated using the Hidden Markov Model Toolkit (HTK) [42] and empirical evidence suggested that a front-end designed to operate in a way that is similar to the human ear and resolve frequencies non-linearly across the audio spectrum and empirical evidence suggests that designing a front-end to operate in a similar non-linear manner improves recognition performance. The Fourier transform based triangular filters are and equally spaced along the mel-scale, which is defined by 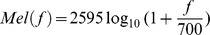 [42]

MFCCs are calculated from the log filterbank amplitudes using the Discrete Cosine Transform 
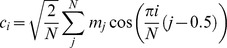
 where *N* is the number of filterbank channels [42].

A sequence of MFCC feature vectors 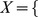 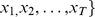 where 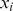 consists of 60 features including MFCCs+Energy+the first and the second derivatives are estimated from the speech recording using a 25 millisecond Hamming window and a frame advance of 10 milliseconds [22].

The Reynolds *et al*. [35] approach to speaker verification based on the Gaussian mixture models was adapted to emotion detection by Dumouchel *et al*. [22]. In this modeling, the probability of observing a feature vector 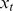 from a given GMM (
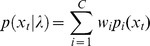
 or alternatively 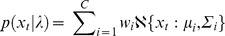) is a weighted combination of 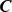 Gaussian densities 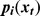 **,** where each Gaussian is parameterized by a mean vector 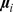 of dimension 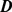 and a covariance matrix 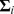 is given by: 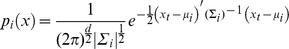



The mixture weights 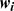 must satisfy the condition 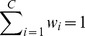. Each emotion class 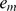 is represented by a single GMM. Each GMM is trained on the data from the same emotion class using the expectation-maximization algorithm [42].

The feature vectors 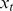 are assumed to be independent; therefore the log likelihood for each emotion model 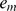 is 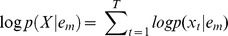. In case of limited data for each class, another approach of training a GMM is to train one large GMM named Universal Background Model, and then adapt this GMM to each emotion data class based on Maximum A Posteriori adaptation. This last training version was the one used in our emotion detection system.

Naïve Bayes rule with equal emotion class weights is used to calculate the maximum likelihood that an utterance 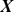 corresponds to the emotion 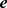. The posterior distribution of each class e given the utterance **X** can be simplified as follows:



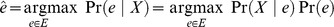


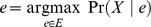


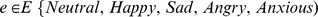



The best five-class Emotion detector overall accuracy at the INTERSPEECH 2009 Emotion Challenge was 41.65% [23] on the FAU Aibo Emotion Corpus consisting of nine hours of German speech samples from 51 children ages 10–13 years, interacting with Sony's pet robot Aibo. The data were annotated by five adult labelers with 11 emotion categories on the word level. Steidl *et al*. [38] found that in most cases three out of five people agreed on the emotional content.

The overall accuracy was 62% (Neutral = 85%, Happy = 70%, Sad = 37%, Angry = 45%, Anxious = 72%) on the emotional corpus of 8,376 speech recordings collected annotated by the labelers. The K-fold cross-validation (K = 10) algorithm was used for model training and test due to the small corpus size. The higher accuracy is hypothesized to be attributed to the closed context of the data collection (participants were explicitly asked for 1 of 5 emotions), and the longer speech segments containing a single emotion. (The mean speech duration was 3.79 seconds.).

**Figure 7:**
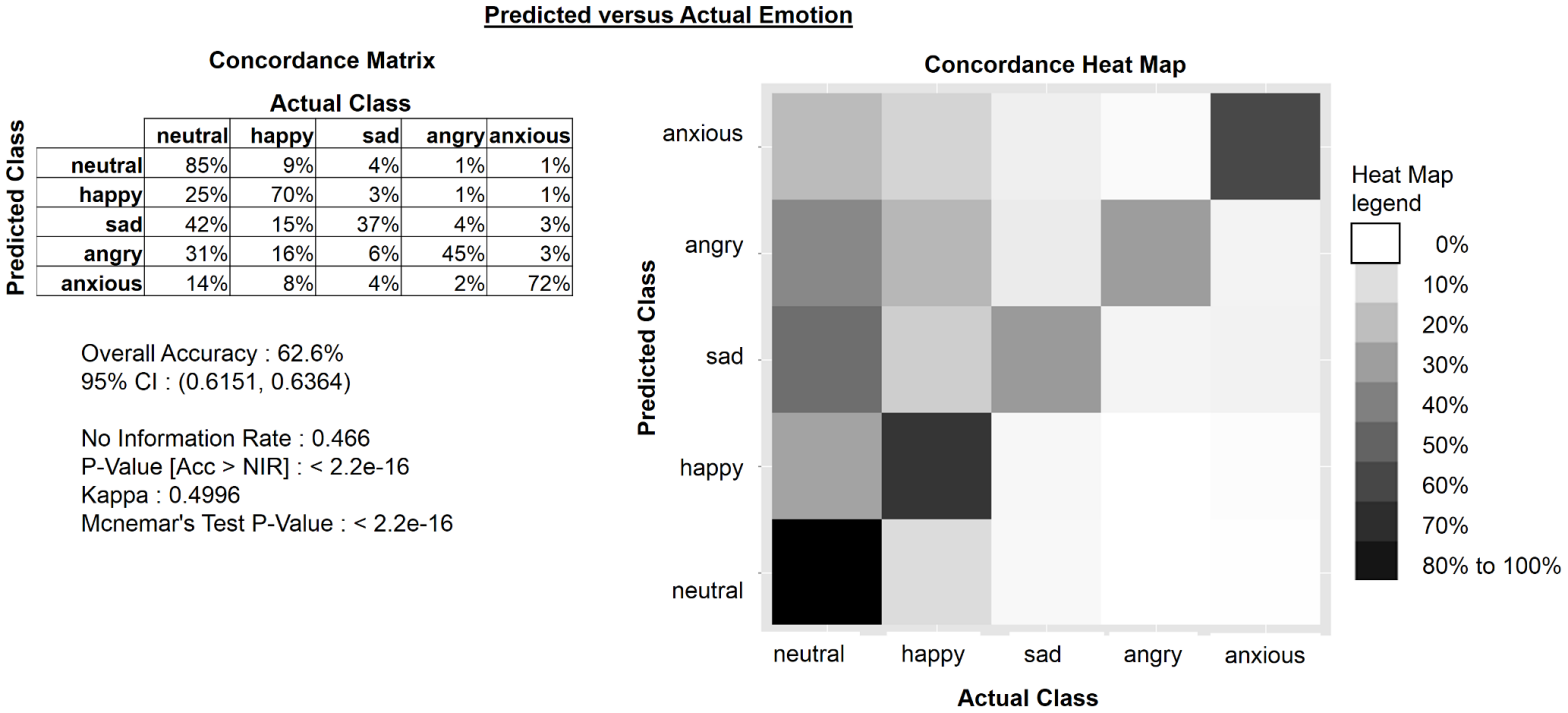
Automatic emotion detector results. The overall accuracy of the speech emotion detector is 62.58% (95% CI: 61.5%–63.6%) The concordance matrix of predicted values from the emotion detector versus the labeled emotion is presented on the left of figure 7. The Diagonal provides the accuracy of each emotional class (predicted emotion = actual emotion). Off-diagonal cells give percentages of false recognition (e.g. anxious accuracy was 72%, with 14% anxious recordings falsely categorized as okay or neutral, 8% falsely categorized as happy, 4% falsely categorized as sad, and 2% falsely categorized as angry). The heat map on the right graphically depicts the concordance matrix with correct predictions on the diagonal (predicted class is flipped upside down).

Figure 7 shows the concordance matrix of predicted values from the emotion detector versus the labeled emotion. The heat map on the right graphically depicts the concordance matrix with correct predictions on the diagonal.

The fused MV and emotion detection classifier provides a high degree of certainty and is at least as accurate as the 3 out of 5 human transcription voting scheme used to annotate the FAU Aibo Emotion Corpus used in the INTERSPEECH 2009 challenge [23], [38]. However, the desired approach is automatic real-time emotion detection in speech without the need for human transcriptions. Efforts at ETS and MIT to improve the accuracy of automatic emotion detection continue. Gaussian distributions underestimate true variance by a factor of (N-1)/N; thus, we will improve the accuracy as we collect more emotional speech data. The current approach consists of extracting MFCCs and energy features from speech, and then classifying these acoustic features to an emotion. There are possibly additional speech features that can be leveraged to increase accuracy [36], [37]. Emotion produces changes in respiration, phonation, articulation, as well as energy. Anger generally is characterized by an increase in mean in fundamental frequency F0, an increase in mean energy, an increase articulation rate, and pauses typically comprising 32% of total speaking time [37]. Fear is characterized by an increase in mean F0, F0 range, and high-frequency energy; an accelerated rate of articulation, and pauses typically comprising 31% of total speaking time. (An increase in mean F0 is evident for milder forms of fear such as worry or anxiety) [37]. Sadness corresponds in a decrease in mean F0, F0 range, and mean energy as well as downward-directed F0 contours; slower tempo; irregular pauses [37]. Happiness produces an increase in mean F0, F0 range, F0 variability, and mean energy; and there may be an increase in high-frequency energy and rate of articulation [37]. Prosodic features such as pitch and energy contours have already been successfully used in emotion recognition [38]. A new, powerful technique for audio classification recently developed at MIT will be investigated for emotion detection. In this new modeling, each recording is mapped into low dimensional space named an I-vector. This new speech representation achieved the best performances on other speech classification domains such as speaker and language recognition [39].

### Emotional Truth

To improve the of overall accuracy of (automatic) emotional ground truth detection, crowd-sourced majority vote (MV) classifiers from anonymous callers and professional transcribers were fused [34] to the current automatic emotion detector (of 62% accuracy). Voters listened to speech recordings and classified the emotion. Anonymous caller vote collection leveraged the empathy collection section of the emotional health Interactive Voice Response dialog. Transcribers labeled speech data using an online tool.

The problem with fusing MV classifiers to the emotion detector is that there is no baseline ground truth to estimate the accuracy of the classification. ReCAPTCHA [43] accuracies on human responses in word transcription are the only empirical data available on the accuracy of crowd-sourced transcription known to these authors. We know of no data regarding the accuracy of a human’s ability to determine the emotion of another human other than Steidl’s 3/5 voter concurrence estimate [38]. ReCAPTCHA [43], used by over 30 million users per day, improves the process of digitizing books by voting on the spelling of words that cannot be deciphered by Optical Character Recognition. The ReCAPTCHA system achieves 99.1% accuracy at the word level; 67.87% of the words required only two human responses to be considered correct, 17.86% required three, 7.10% required four, 3.11% required five, and only 4.06% required six or more. We assumed ReCAPTCHA word transcription accuracies as an approximation to emotion MV accuracy and calculated the “certainty” or accuracy of the MV result from a regression model based on ReCAPTCHA human response agreement.

ReCAPTCHA_*certainty_fac*tor = 0.13768+0.16982×(# *human responses*).

Thus, two humans in agreement will result in a certainty factor of 47.7%; five humans in agreement results in a certainty factor of 98.7%, and six votes or more produces a certainty factor of 100%.

Given the independent categorical variable 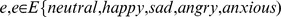, we computed a count of 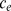 of votes for each 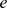, and the total count 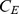 for all emotions where 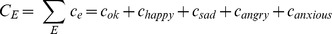. The MV estimate for 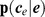 is the division of 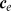 by 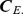 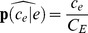
_._

The fused emotional ground truth classifier for emotion 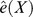, where 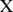 is the audio recording, is given by:



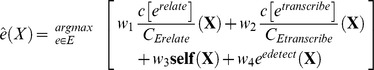


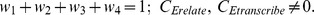



The score for 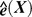 is the confidence measure, 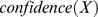. The “certainty” or predicted accuracy of 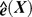 is estimated by:



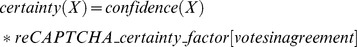



**Figure 8:**
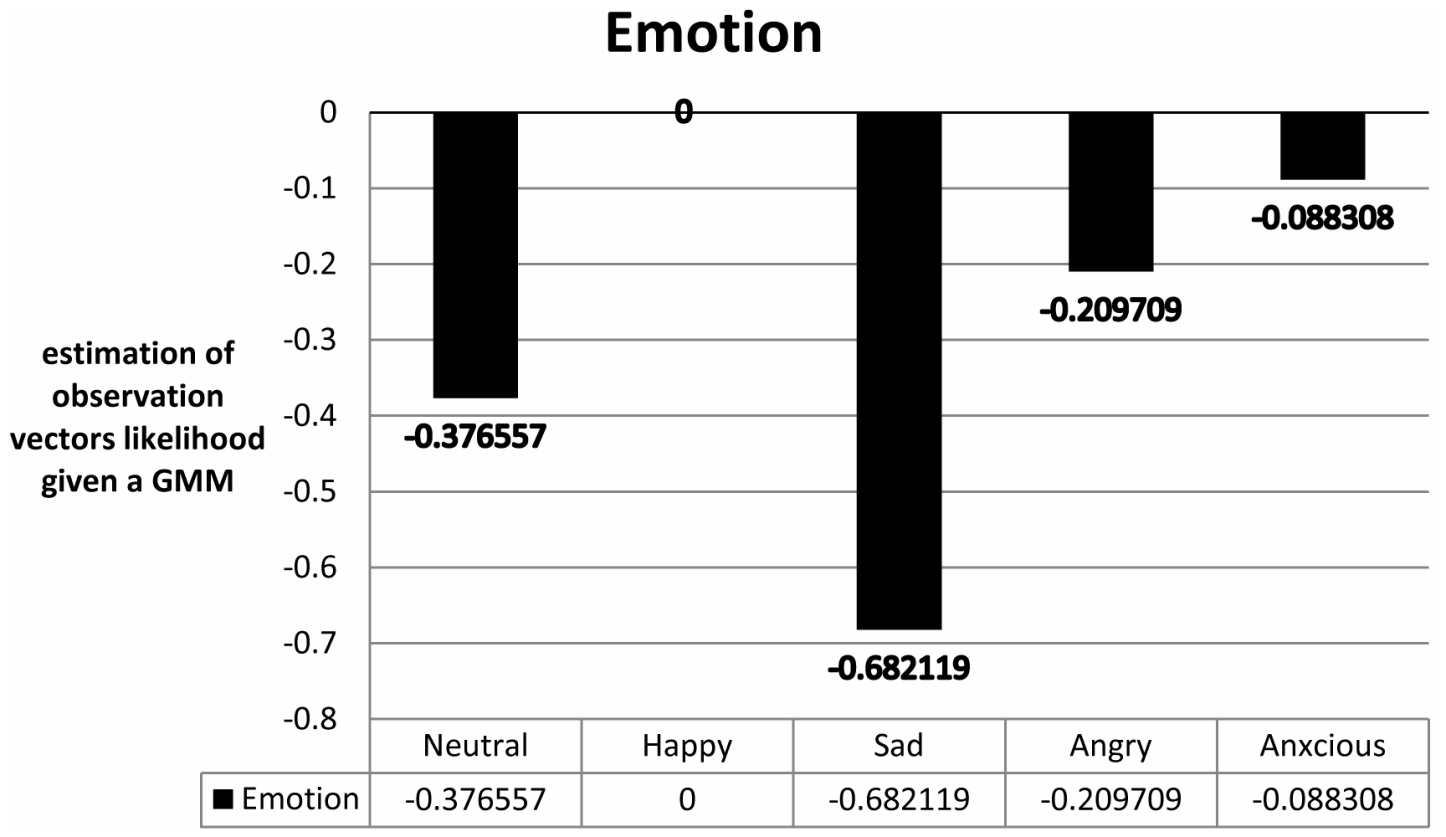
Example of automatic emotion detection likelihood estimation. Naïve Bayes with equal emotion class weights is used to calculate the maximum likelihood that an utterance X corresponds to the emotion e. In this example the automatic emotion detector classified the speech recording as Happy, with a likelihood estimation (score) of 0 (the higher the score, the more likely the classification).

In the example of Figure 8, the automatic emotion detector classified the speech recording as Happy, with a likelihood estimation (score) of 0. The score difference between Happy versus Neutral, Sad, and Angry indicated good separation in the activation-evaluation emotional space, as shown in the scores on the columns of the graph. There was less separation between Happy and Anxious.

In Table 2, the vote sources from the phone call relate, transcription, self-assessment, and emotion detection are in agreement that the recording contains the emotion Happy.

**Table 2:** Example of majority-vote sources.

Vote Source	Total Votes	Neural	Happy	Sad	Angry	Anxious
**Phone Call Relates**	1	0	1	0	0	0
**Transcriptions**	8	1	7	0	0	0
**Self Assessment**			1			
**Emotion Detection**			1			

Applying the fused emotional ground truth classifier, we computed the probabilities of each emotion as depicted in Table 3. The probability of Happy is highest with a confidence measure of 95%. The certainty of Happy is 95% * 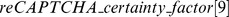 = 95%.

**Table 3:** Example of calculation of emotion from four sources.

Probability	relate w1*(c/C)	Transcriber w2*(c/C)	Self w3(c)	Edetect w3(c)	Confidence (∑)
P(X|neutral)	0.3* (0/1) = 0	0.4*(1/8) = 0.05	0.1*(0) = 0	0.2*(0) = 0	0.05
P(X|happy)	0.3*(1/1) = 0.3	0.4*(7/8) = 0.35	0.1*(1) = 0.1	0.2*(1) = 0.2	0.95
P(X|sad)	0.3*(0/1) = 0	0.4*(0/8) = 0	0.1*(0) = 0	0.2*(1) = 0	0
P(X|angry)	0.3*(0/1) = 0	0.4*(0/8) = 0	0.1*(0) = 0	0.2*(1) = 0	0
P(X|anxious)	0.3*(0/1) = 0	0.4*(0/8) = 0	0.1*(0) = 0	0.2*(1) = 0	0
argmax(X|e)	happy with confidence = 0.95				


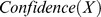
, the ratio of votes in agreement, has been established as an indication of emotion expressiveness in terms of confusability and flat affect rather than 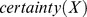. The number of votes collected across the emotion corpus varies, following a normal distribution, and it would be unfair to penalize a patient’s measurement of expressiveness due to number of votes in agreement.

### Statistical Analyses

Generalized Linear Mixed Model (GLMM) regression analyses [44]–[50] were performed using the glmer() function in the R package lme4 [44] to determine if there were significant differences in emotional truth, self-awareness, empathy, and expressiveness across group, gender, and language. Call completion rates were explored across groups as a possible indicator of apathy or relapse. Statistically significant results are presented in the Results section.

The data collected were multilevel, with emotional data at the micro-level and participants at the macro-level. The number of participants in each population group and emotional data per participant was unbalanced. Aggregated Ordinary Least Squares regression analysis is inaccurate in this case, as 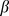 coefficients are estimated as a combination of 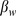 (within-group) and 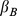 (between-group). Hierarchical Linear Models or Mixed-Effects models are more appropriate for representing hierarchical clustered dependent data. Mixed-effects models incorporate fixed-effects parameters, which apply to an entire population; and random effects, which apply to observational units. The random effects represent levels of variation in addition to the per-observation noise term that is incorporated in common statistical models such as linear regression models, generalized linear models, and nonlinear regression models [44]–[50].

Each 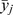 for 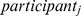 gives some information towards calculating the overall population average 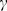. Some 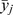 provide better information than others (i.e., 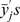 from a larger observation cluster 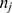 will give better information than a 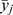 from a smaller observation cluster 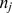). How do you weigh the 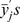 in an optimal manner? *Answer: weigh by the inverse of their variance.* All observations then contribute to the analysis, including participants who have as few as one observation, since the observations are inversely weighted by within-group variance [45].

The simplest example to move from Ordinary Least Squares to Hierarchical Linear Models is the one regression coefficient problem 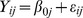 where 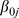 is the intercept (population average), and 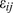 is the residual effect of micro-unit 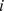 within macro-unit 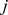 [48]. Applying Hierarchical Linear Models proceeds as follows:

Level 1 model: 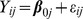


Level 2 model: 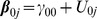


Mixed-model (Hierarchical Linear Model): 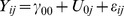 where 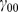 is the fixed effect, and 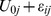 are the random effects.

The overall variance 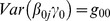. The variance for 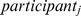 is given as 
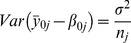
 But this does not tell us how to apply the patient’s variance 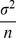 as an estimator of 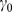 = 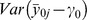. We need to calculate: 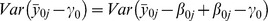


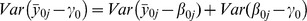


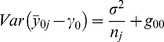



The overall population average is 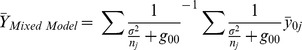


Intraclass Correlation coefficient: 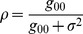



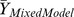
 is an optimized estimator of overall mean that takes into account, in an optimal way, information contained in each participant’s mean. Weight contribution from each participant depends on 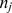 and 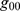. Thus a participant with 100 samples will contribute more than a participant with 1 sample, but the 1 sample cluster can still be leveraged to improve the overall estimate.

Complexity increases as coefficients are added. A one-level, two-regression-coefficient Ordinary Least Squares model is formulated as: 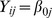
+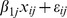. The intercepts 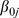 as well as the regression coefficients 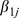 are group-dependent. To move to a mixed-effect model, the group-dependent coefficients can be divided into an average coefficient and the group-dependent deviation 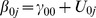 and 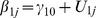


Substitution gives model: 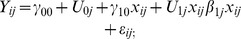


Fixed effects: 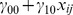


Random effects: 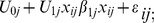


Goodness-of-fit for Hierarchical Linear Models leverage the Akaike information criterion, Bayesian information criterion, Log Likelihood, and Deviance measures produced by glmer() rather than the classic Ordinary Least Squares 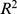. Snijders [45] and Boroni [50] prefer the Deviance measurement. The difference in deviance between a simpler and a more complex model approximates a 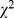 (chi-squared) distribution with the difference in number of parameters as df’s. Improvement is significant (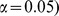 if the deviance difference is larger than the parameter difference. In emotional data analysis, single factor models were compared against the “null” model. Multifactor analysis was not possible due to insufficient data.

## Results

**Figure 9:**
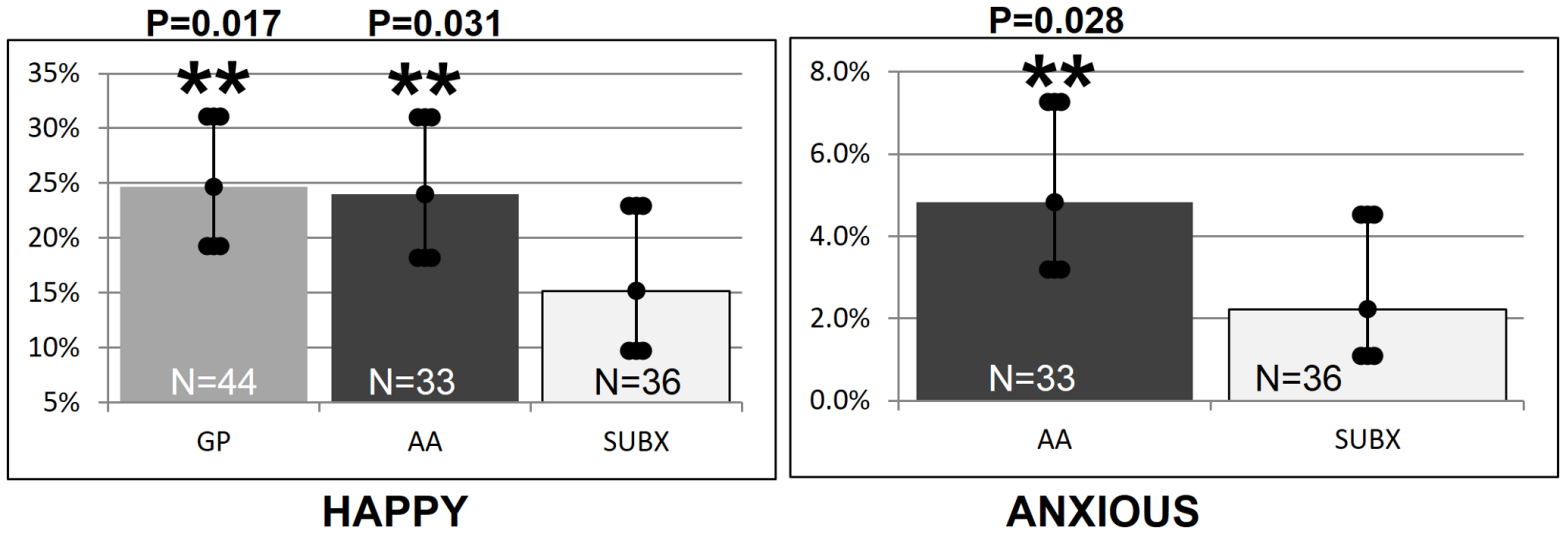
Significant emotion differences across groups. Statistical analyses of emotions showed that SUBX patients had a lower probability of being happy (15.2%; CI: 9.7–22.9) than both the GP (p = 0.0171) (24.7%; CI: 19.2–31.0) and AA groups (p = 0.031) (24.0%; CI: 18.2–31.0). However, AA members had over twice the probability of being anxious (4.8%; CI: 3.2–7.3) than SUBX patients (p = 0.028) (2.2%; CI: 1.1–4.5).

Statistical analyses of emotions showed that SUBX patients had a lower probability of being happy (15.2%; CI: 9.7–22.9) than both the GP (p = 0.171) (24.7%; CI: 19.2–31.0) and AA groups (p = 0.031) (24.0%; CI: 18.2–31.0). However, AA members had over twice the probability of being anxious (4.8%; CI: 3.2–7.3) than SUBX patients (p = 0.028) (2.2%; CI: 1.1–4.5) (Figure 9).

**Figure 10:**
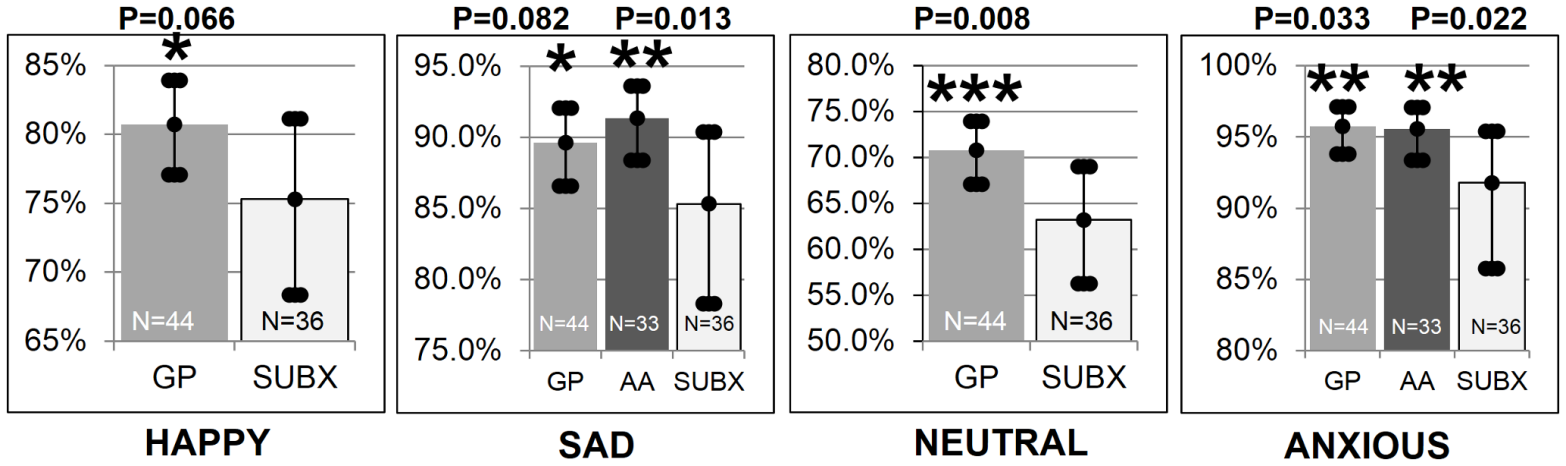
Significant differences in emotional self-awareness across groups. Figure 10 shows that the SUBX patients tended to be less self-aware of happiness (75.3%; CI:68.4–81.1) than the GP group (p = 0.066) (78.8%; CI: 76.6–80.8); less self-aware of sadness (85.3%; CI:78.3–90.3) than AA members (p = 0.013) (91.3%; CI: 87.4–93.6) and tended to be less self-aware of sadness than the GP (p = 0.082) (89.6%; CI: 87.8–91.2); less self-aware of their neutral state (63.2%; CI:57.0–69.0) than the GP (p = 0.008) (70.7%; CI:67.3–74.0) and AA members (p = 0.022) (71.7%; CI: 68.9–74.3); and less self-aware of their anxiety (91.8%; CI: 85.8–95.4) than the GP (p = 0.033) (95.8%; CI:93.4–97.1) and AA members (p = 0.022) (95.6%; CI:93.4–97.1).

Figure 10 shows that the SUBX patients tended to be less self-aware of happiness (75.3%; CI:68.4–81.1) than the GP group (p = 0.066) (78.8%; CI: 76.6–80.8); less self-aware of sadness (85.3%; CI:78.3–90.3) than AA members (p = 0.013) (91.3%; CI: 87.4–93.6) and tended to be less self-aware of sadness than the GP (p = 0.082) (89.6%; CI: 87.8–91.2); less self-aware of their neutral state (63.2%; CI:57.0–69.0) than the GP (p = 0.008) (70.7%; CI:67.3–74.0); and less self-aware of their anxiety (91.8%; CI: 85.8–95.4) than the GP (p = 0.033) (95.8%; CI:93.4–97.1) and AA members (p = 0.022) (95.6%; CI:93.4–97.1).

**Figure 11:**
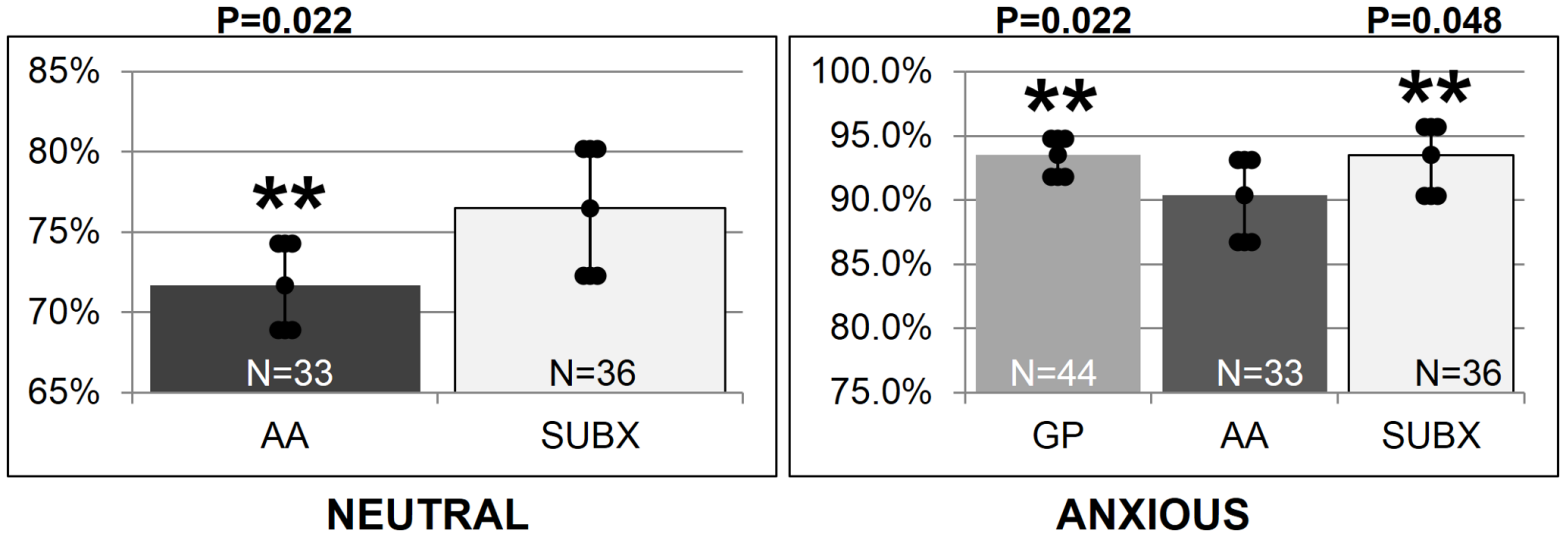
Significant differences in emotional empathy across groups. Interestingly, and as can be seen in Figure 11, the SUBX patients were more empathic to the neutral emotion state (76.5%; CI: 72.3–80.2) than AA members (p = 0.022) (71.7%; CI: 68.9–74.3). AA members were less empathic to anxiety (90.4%; CI: 86.7–93.1) than the GP (p = 0.022) (93.5%; CI: 91.8–94.8) and SUBX patients (p = 0.048) (93.5%; CI: 90.3–95.7).

Interestingly, and as can be seen in Figure 11, the SUBX patients were more empathic to the neutral emotion state (76.5%; CI: 72.3–80.2) than AA members (p = 0.022) (71.7%; CI: 68.9–74.3). AA members were less empathic to anxiety (90.4%; CI: 86.7–93.1) than the GP (p = 0.022) (93.5%; CI: 91.8–94.8) and SUBX patients (p = 0.048) (93.5%; CI: 90.3–95.7).

**Figure 12:**
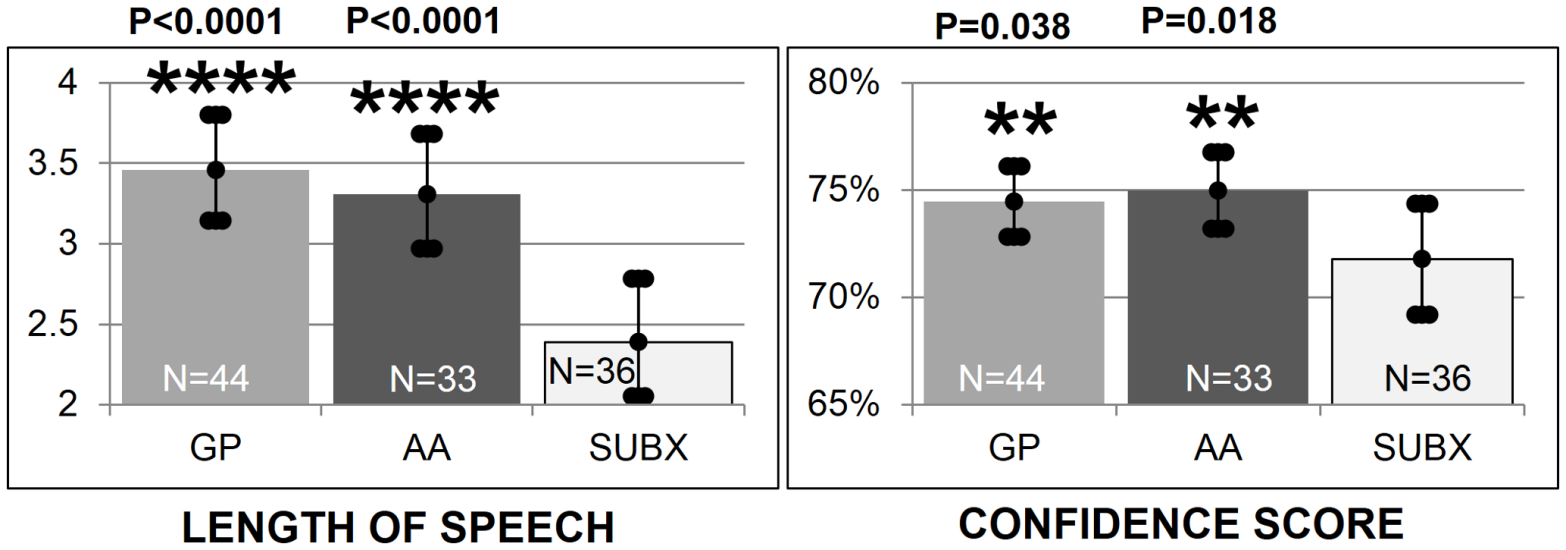
Significant differences in emotional expressiveness across groups. Figure 12 shows that the SUBX group had significantly less emotional expressiveness, as measured by length of speech, than both the GP group and the AA group (p<0.0001). It may be difficult to determine the emotion of SUBX patients, both by humans and by the automatic detector, due to flatter affect. The average audio response to “How are you feeling?” was (3.07 seconds; CI: 2.89–3.25). SUBX patients’ responses were significantly shorter (2.39 seconds; CI: 2.05–2.78)) than both the GP (p<0.0001) (3.46; CI: 3.15–2.80) and AA members (p<0.0001) (3.31; CI: 2.97–3.68). In terms of emotional expressiveness as measured by confidence scores, the SUBX group also showed significantly lower scores than both the GP and the AA groups. There was significantly less confidence in SUBX patients’ audio responses (72%; CI: 0.69–0.74) than the GP (p = 0.038) (74%; CI: 0.73–0.76) and AA members (p = 0.018) (75%; CI: 0.73–0.77).

Figure 12 shows that the SUBX group had significantly less emotional expressiveness, as measured by length of speech, than both the GP group and the AA group (p<0.0001). It may be difficult to determine the emotion of SUBX patients, both by humans and by the automatic detector, due to flatter affect. The average audio response to “How are you feeling?” was (3.07 seconds; CI: 2.89–3.25). SUBX patients’ responses were significantly shorter (2.39 seconds; CI: 2.05–2.78)) than both the GP (p<0.0001) (3.46; CI: 3.15–2.80) and AA members (p<0.0001) (3.31; CI: 2.97–3.68). In terms of emotional expressiveness as measured by confidence scores, the SUBX group also showed significantly lower scores than both the GP and the AA groups. There was significantly less confidence in SUBX patients’ audio responses (72%; CI: 0.69–0.74) than the GP (p = 0.038) (74%; CI: 0.73–0.76) and AA members (p = 0.018) (75%; CI: 0.73–0.77).

It is noteworthy that in this sample we also observed the following trends regarding gender: Women were less aware of sadness (87.5%; CI: 84.1–90.3) than men (p = 0.053) (91.0%; CI: 87.4–93.6); women had more empathy towards anxiety (93.7%; CI: 92.1–94.9) than men (p = 0.08) (91.8%; CI: 89.2–93.9); and women had more empathy towards anger (95.1%; CI: 91.8–94.8) than men (p = 0.099) (94.1%; CI: 86.7–93.1). Additionally, English people were less neutral (40.5%; CI: 33.9–47.5) than French people (p = 0.05) (49.3%; CI: 40.3–58.3), and French people were more emphatic to anger (95.4%; CI: 93.4–96.8) than English people (p = 0.02) (93.0%; CI: 91.0–94.6).

## Discussion

Call rate analysis provides interesting results. Kaplan-Meier survival estimate is 56% that a participant will complete a 60 day trial. Participants answered 41% of emotion collection calls on average. SUBX answered significantly fewer calls (18.6%) as compared to GP (56.4%) and AA (49.3%).

**Figure 13:**
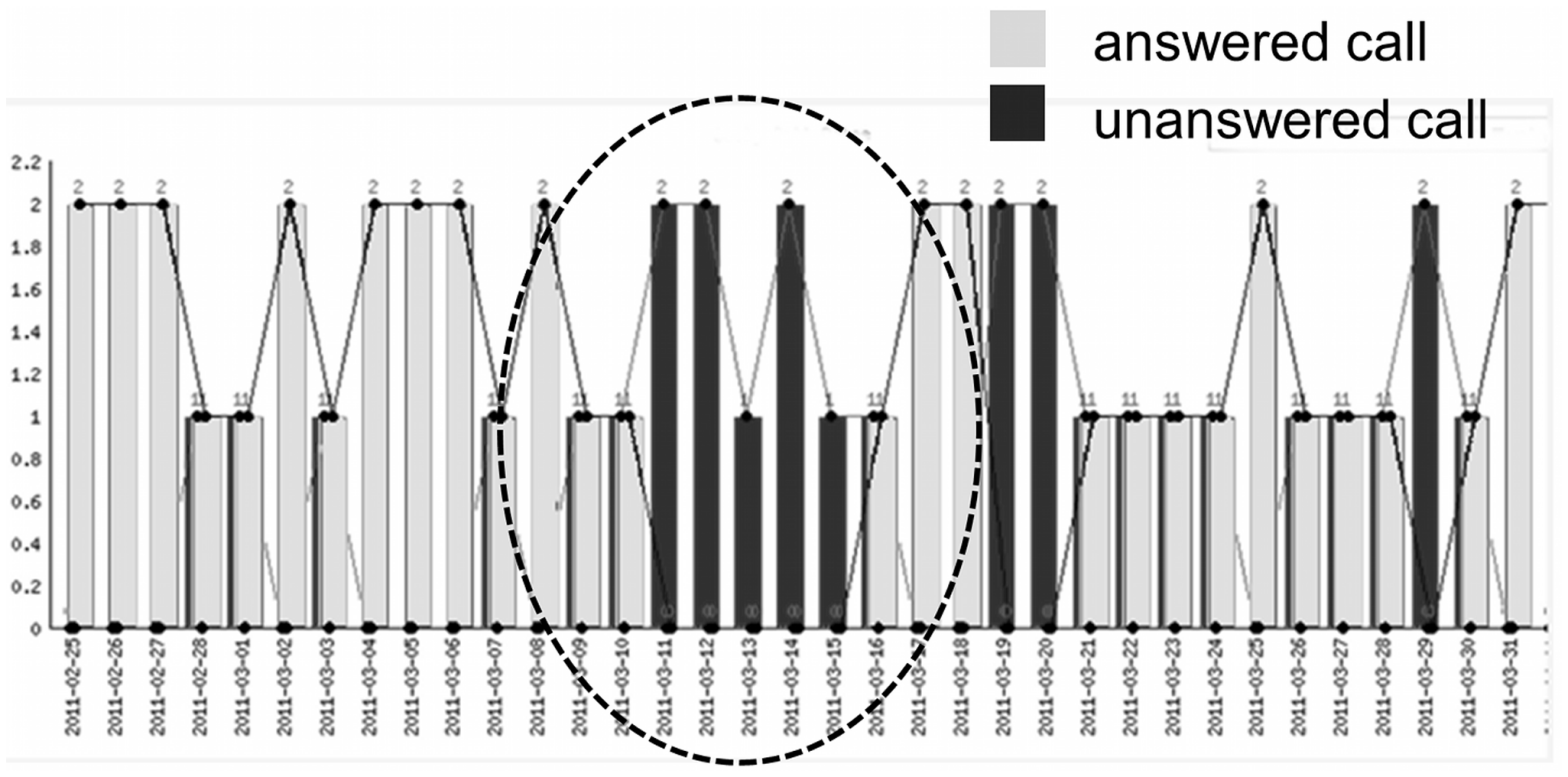
SUBX patient call rate with period of missed calls. This figure captures daily call rates for an actual SUBX patient. The Y axis indicates the number of calls made to a patient per day (X axis). Successful calls, where a momentary emotional state is registered, are represented in light grey. Unsuccessful calls, where there was no answer or the answering machine responded, are represented in dark grey. There is a period from 2011-03-11 (March 11, 2011) thru to 2011-03-15 where the SUBX patient did not answer their phone. In the worst case this could be an indication of relapse or isolation due to depression. In the best case this could coincide with the patient being away from their home or cell phone, or simply apathy towards the IVR system. In a clinical implementation, a notification could be automatically sent to the therapist or case worker for follow-up.

There is an inference that SUBX patients also may covertly continue to misuse licit and illicit drugs during treatment, timing their usage to avoid urine detection. SUBX patients were tested on a scheduled monthly basis. In urine the detection time of chronic opioid users is 5 days after last use [51]. A patient may correctly anticipate that once a urine specimen has been obtained in a certain calendar month, no further specimen will be called for until the next month. Indeed, we have heard from many patients that they understand this only too well–that they have a ‘‘free pass’’ until the next month” [51]. In Figure 13, it is evident for the sample SUBX patient that there was a lapse in answering calls from March 11 through the 15^th^. The monitoring capability of the toolkit provides a mechanism to automatically send an email or text message on this unanswered call condition or mood conditions (e.g. consecutive days of negative emotions) to alert professionals for intervention.

In subsequent follow-up studies underway, our laboratory is investigating both compliance to prescribed treatment medications and abstinence rates in a large cohort of SUBX patients across six Eastern Coast States and multiple addiction treatment centers utilizing a sophisticated Comprehensive Analysis of Reported Drugs (CARD) ™ [52].

The long-term SUBX patients in the present study showed a significantly flat affect (p<0.01), having less self-awareness of being happy, sad, and anxious compared to both the GP and AA groups. This motivates a concern that long-term SUBX patients due to a diminished ability to perceive “reward” (an anti-reward effect [16]) including emotional loss may misuse psychoactive drugs, including opioids, during their recovery process. We are cognizant that patients on opioids, including SUBX and methadone, experience a degree of depression and are in some cases prescribed anti-depressant medication. The resultant flat affect reported herein is in agreement with the known pharmacological profile of SUBX [53].

We did not monitor the AA group participants in terms of length of time in recovery in the AA program and this may have an impact on the results obtained. If the participants in the AA group had been in recovery for a long time the observed anxiousness compared to the SUBX group may have been reduced. However, it is well-known that alcoholics are unable to cope with stress and this effect has been linked to dopaminergic genes [54].

We know from the neuroimaging literature that buprenorphine has no detectible effect on the prefrontal cortex and cingulate gyrus [55] regions thought to be involved with drug abuse relapse [56], [57]. We must then, consider the potential long-term effects of reduced affect attributed to SUBX. Blum *et al.* [16] proposed a mechanism whereby chronic blockade of opiate receptors, in spite of only partial opiate agonist action, could block dopaminergic activity inducing anti-reward and potentially result in relapse.

It is well known that individuals in addiction treatment and recovery clinics tend to manipulate and lie not only about the licit and or illicit drugs they are misusing, but also their emotional state. Comings *et al.* [57] identified two mutations (G/T and C/T that are 241 base pairs apart) of the dopamine D2 receptor (DRD2) gene haplotypes by using an allele specific polymerase chain reaction. These haplotypes were found in 57 of the Addiction Treatment Unit subjects and 42 of the controls. Subjects with haplotype 1 (T/T and C/C) tended to show an increase in neurotic, immature defense styles (lying) a decrease in mature defense styles compared to those without haplotype 1. Each of the eight times that the subscale scores in the questionnaire were significant for haplotype 1 versus non-1, those with haplotype 1 were always those using immature defense styles. There results suggest that one factor controlling defense styles is the DRD2 locus. Differences between mean scores of controls and substance abuse subjects indicated that other genes and environmental factors also play a role. This fact provides further impetus to repeat the experiments on methadone, heroin and opioid abstinent controls using this more objective methodology and compare these potential new results with our current Suboxone ™ data.

Despite its disadvantages, SUBX is available as a treatment modality that is effective in reducing illicit opiate abuse while retaining patients in opioid treatment programs, and until a real magic bullet is discovered, clinicians will need to continue to use SUBX. Based on these results, and future research into strategies that can reduce the fallibility of journaling and increase reliability in the detection of emotional truth we hope to more accurately determine the psychological status of recovering patients. We recommend that combining expert, advanced urine screening, known as Comprehensive Analysis of Reported Drugs (CARD) [52] with accurate determination of affective states (“true ground emotionality”) could counteract the lack of honesty in clinical dialogue and improve the quality of interactions between patients and clinicians. Since we have quantified the emotionality of long term SUBX patients to be flat, we encourage the development of opioid maintenance substances that will provide up-regulation of reward neurotransmission ultimately resulting in the normalization of one’s mood. To reiterate until we perform required appropriate opioid abstinent controls any interpretation of these results must remain suspect.
