# Correction: Long Term Suboxone™ Emotional Reactivity As Measured by Automatic Detection in Speech

**DOI:** 10.1371/annotation/be0b3a26-c1bc-4d92-98c1-c516acc8dcf2

**Published:** 2013-08-15

**Authors:** Edward Hill, David Han, Pierre Dumouchel, Najim Dehak, Thomas Quatieri, Charles Moehs, Marlene Oscar-Berman, John Giordano, Thomas Simpatico, Debmalya Barh, Kenneth Blum

Author Debmalya Barh was omitted from the original submission. The full list of authors and affiliations can be found below:

Edward Hill^1^

David Han^2^

Pierre Dumouchel^1^

Najim Dehak^3^

Thomas Quatieri^4^

Charles Moehs^5^

Marlene Oscar-Berman^6^

John Giordano^7^

Thomas Simpatico^8^

Debmalya Barh^13^

Kenneth Blum^7,8,9,10,11,12,13^

**1** Department of Software and Information Technology Engineering, École de Technologie Supérieure - Université du Québec, Montréal, Québec, Canada

**2** Department of Management Science and Statistics, University of Texas at San Antonio, San Antonio, Texas, United States of America

**3** Computer Science and Artificial Intelligence Laboratory, Massachusetts Institute of Technology, Cambridge, Massachusetts, United States of America

**4** Lincoln Laboratory, Massachusetts Institute of Technology, Lexington, Massachusetts, United States of America

**5** Occupational Medicine Associates, Watertown, New York, United States of America

**6** Departments of Psychiatry, Neurology, and Anatomy and Neurobiology, Boston University School of Medicine, and Boston Veteran Affaires Healthcare System, Boston, Massachusetts, United States of America

**7** G & G Holistic Health Care Services, LLC., North Miami Beach, Florida, United States of America

**8** Global Integrated Services Unit University of Vermont Center for Clinical and Translational Science, College of Medicine, Burlington, Vermont, United States of America

**9** Department of Psychiatry and McKnight Brain Institute, University of Florida, College of Medicine, Gainesville, Florida, United States of America

**10** Dominion Diagnostics, LLC, North Kingstown, Rhode Island, United States of America

**11** Department of Clinical Neurology, Path Foundation New York, New York, United States of America

**12** Department of Addiction Research and Therapy, Malibu Beach Recovery Center, Malibu Beach, California, United States of America

**13** Institute of Integrative Omics and Applied Biotechnology, Nonakuri, Purba Medinipur, West Bengal, India 

